# Long versus short cephalomedullary femoral nail for treatment of unstable intertrochanteric fractures: A single-blinded randomized controlled study

**DOI:** 10.1051/sicotj/2025065

**Published:** 2026-04-15

**Authors:** Abdelrahman Amir AbouelEla, Haytham Abdel-Azim, Ahmed Sayed Kotb

**Affiliations:** Orthopaedic Surgery Department, Faculty of Medicine, Ain Shams University 38 Abbassia 11591 Cairo Egypt

**Keywords:** Intertrochanteric fractures, Cephalomedullary femoral nails, AO/OTA classification, Harris hip score

## Abstract

*Background*: ITF are extracapsular proximal femoral fractures that occur in both younger and older populations, with a higher prevalence among females. They account for most hip fractures, reaching 44.1%. The Elderly are at risk with an increased first-year mortality risk reaching up to 30%. This research aimed to compare the functional outcomes, effectiveness, and safety profile of long as opposed to short cephalomedullary nails (CMNs) in the management of unstable ITF in elderly individuals aged >60 years. *Methods*: This single-blinded randomized controlled research was carried out on 30 participants aged >60 years old, both sexes, with unstable ITF. Participants were categorized into two groups (GPs): GP A: had a long cephalomedullary nail (LCMN), and GP B: had short cephalomedullary nail. *Results:* Mean hospital stay length, period of surgery, operative blood loss, and the incidence of transfusion requirements were higher in GP A, yet no significant difference was observed. Functional outcomes, union and complication rates were comparable between the two GPs. *Conclusions*: Irrespective of the length, CMNs are suitable for the treatment of unstable ITF, aiming to achieve early mobility and satisfactory functional outcome. Further large-sampled RCTs need to be conducted comparing both GPs based on more recent CT-based classification systems with osteoporosis considered.

## Introduction

Intertrochanteric femoral fractures (ITF) represent extra-capsular fractures extending between the greater (GT) and lesser trochanters (LT) [1]. Due to its trabecular nature, the elderly are subjected to osteoporotic fractures with an increased first-year mortality risk reaching up to 30% compared to femoral neck fractures [2].

ITF accounts for most hip fractures, reaching up to 44.1% [3]. They are treated with internal fixation, whether by dynamic hip screws (DHS) or cephalomedullary nails (CMN), with the latter being the most frequently used, for both stable and unstable fractures [4].

ITFs are classified according to their stability into stable and unstable. Stability is assessed by an intact calcar femoral, decreased lateral wall thickness below 20.5 mm, subtrochanteric extension, and the reverse oblique subtype [5, 6].

CMN are classified into long and short, with a length of 250 mm as a cut-off, and short nails not crossing the femoral isthmus [7]. Across the literature, LCMN and SCMN were comparable biomechanically, with LCMN having the theoretical advantage of increased stability in ITF with subtrochanteric extension and a lower incidence of periprosthetic diaphyseal fractures. However, nail-femur curvature radius mismatch might lead to anterior cortex perforation, which could be avoided by proper nail size and posterior entry point [8].

The dimensions of the LT may also influence the choice of CMN, as fracture fragments located inferior to the base of the LT by 40 mm may render the SCMN to fail with subsequent distal cortical plastic deformation [9]. In contrast, SCMN had shorter surgical duration, minimized blood loss, decreased transfusion requirements, and decreased costs [10].

This study aimed to compare the operative time, functional outcome, efficacy, and safety profile of LCMN and SCMN in treating unstable ITF fractures in patients over 60 years old.

## Materials and methods

This single-blinded randomized research was carried out on 30 participants aged >60 years old, both sexes, with unstable ITF. The investigation was executed from December 2023 to December 2024, after approval from the Ethical Committee of Ain Shams University Hospitals, Cairo, Egypt (FMASU MS 699/2023). Retrospectively registered in PACTR (PACTR202506720274898) due to logistical causes. All participants provided written informed consent before enrolment.

Exclusion criteria were patients with stable intertrochanteric fractures, unstable intertrochanteric fractures with subtrochanteric extension, associated hip pathology, bedridden or immobile patients, pathological fractures secondary to malignant disease, previous ipsilateral hip surgeries, and extreme anteroposterior (AP) femoral bowing or deformity.

### Randomization and blindness

Patients were randomized through a computer-generated random number table to assign participants with ITF into two groups: GP A Underwent treatment involving an LCMN, whereas those in GP B received a SCMN.

A thorough clinical evaluation and appropriate radiological investigations [X-rays and computed tomography (CT)] were performed for all participants.

Preoperative evaluation: modified Harris hip score (MHHS) was applied to estimate the baseline functional status and multiplied by 1.1 to calculate the pre-injury HHS [11].The Barthel index (BI), an indicator for the activity of daily living, was used to assess the independence of the patients before the fracture.

### Surgical technique

**Figure 1:**
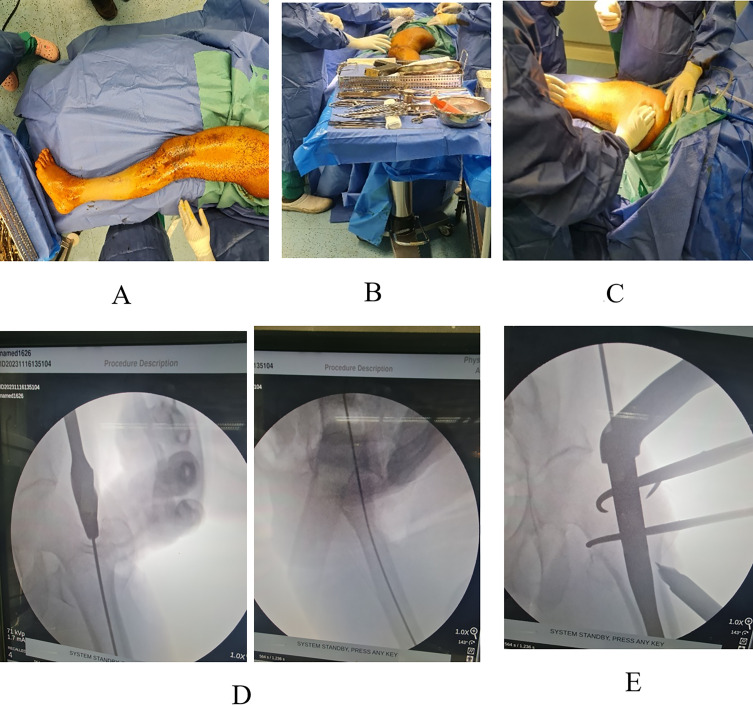
Surgical technique.

Patients underwent spinal anesthesia and were positioned laterally with the affected limb on top. A trial of reduction was done to correct the anatomical alignment, angulation, and version of the proximal femur using fluoroscopy (Figure 1A). The whole affected lower limb was then sterilized with povidone iodine and draped from the iliac crest down toward the foot. Intravenous antibiotic; Cefazolin® is given 30 min before incision time in weight-adjusted dosing. The main surgeon stands facing the posterior aspect of the thigh with the assistant standing on the opposite side (Figure 1B). The GT is palpated (Figure 1C), and then a 5 cm incision proximal to the tip of the GT is made with a slight posterior inclination. Fractures were either closed or openly reduced. Reduction was aided by the use of bone hooks, pointed clamps, hohmans, and manual manipulation (Figure 1D).

After fracture reduction, an entry point is purchased using a cannulated awl just medial to the GT tip per company instructions (Figure 1E). Serial Reaming was done till reaching a suitable canal diameter, followed by nail placement, whether by LCMN or SCMN. GT fractures were addressed by either a cortical 6.5 mm screw or direct repair with ethibond® size 2 sutures. All nails were provided by ORTHOMED ® Company with SCMN length ranging from 180 to 240 mm and LCMN length ranging from 340 to 420 mm. Regarding the width, all nails, whether SCMN or LCMN, ranged in width from 10 mm to 13 mm according to the reamed canal diameter. All lag screws inserted were 10.5 mm in diameter and ranged from 90 to 115 mm in length. In 22 out of 30 cases, anti-rotation screws were utilized with a diameter of 6.5 mm and a length ranging from 80 to 105 mm. The remaining eight cases used endcaps instead of an anti-rotation screw. 4.8 mm Distal locking screws locked the nail distally.

Drains were utilized according to the amount of blood oozing. Total operative time was measured from the incision time to the start of skin closure. Blood loss was measured from suction devices, collected blood, and soaked dressings.

*Postoperative management*: prophylactic novel anticoagulants were offered to Guard against deep vein thrombosis. Intravenous Antibiotics were continued 48 h post-procedure to guard against infection, and appropriate analgesia was provided for all patients. Partial weight bearing was individualized based on patient factors, surgeon judgment, fracture pattern, and fixation quality. patients were discharged on oral Antibiotics after a Satisfactory wound status.

X-rays were done postoperatively with follow-up imaging at 3 weeks, 3 months, and 6 months intervals, monitoring for signs of union; assessed by callus formation and fracture line disappearance in 3 out of 4 cortices in AP and lateral views, and signs of complications. Functional outcomes were calculated using HHS and BI at 3 weeks, 3 months and 6 months.

The HHS is used to quantify functional outcome, with higher scores reflecting better clinical results. Scores were recorded and calculated during follow-up visits. The maximum attainable score is 100, and outcomes are interpreted as follows: scores <70 were considered indicative of a poor outcome; scores 70–80 were classified as fair, 80–90 as good, and 90–100 as excellent.

### Outcome evaluation

Intraoperative variables, which compare operative duration, intraoperative blood loss, blood transfusion rate, and technical calculations including tip apex distance (TAD), nail length and width, reduction type, and the use of antirotation or endcap. Post-operative outcome, which includes duration of inpatient hospitalization and radiographic union, functional outcomes (HHS and BI) and complications covering radiographic parameters (non-union, implant failure, periprosthetic fracture, varus collapse, heterotopic ossification) and clinical outcomes (time to weight bearing, fracture related infection (FRI), hip pain, mortality), used to compare safety and efficacy between GPs.”

## Statistical analysis

It was performed using SPSS version 26 (IBM Corp., Chicago, IL, USA). Quantitative variables were shown as mean ± standard deviation (SD), and the relation between GPs was assessed using the unpaired Student’s *t*-test. Categorical variables were shown as frequencies and percentages, and interpreted using the Chi-square test or Fisher’s exact test. A *p*-value of <0.05 was considered statistically significant.

## Results

**Figure 2:**
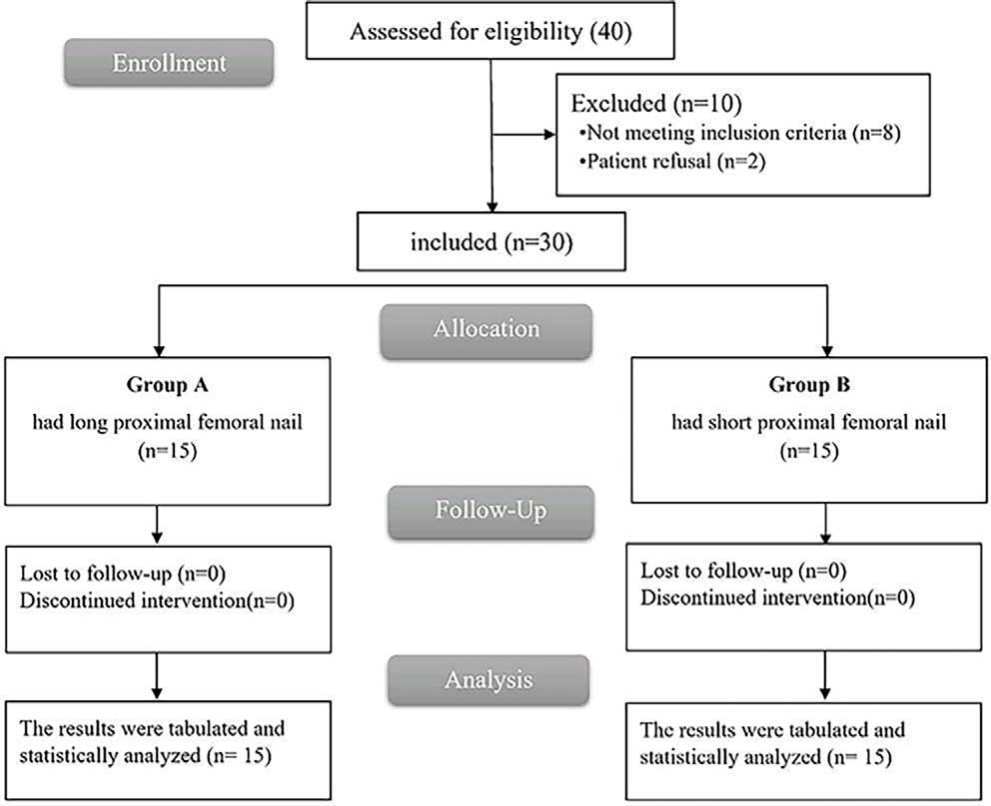
CONSORT flowchart of the enrolled patients.

In this research, 40 participants were initially screened for inclusion criteria. Of these, 8 participants failed to fulfill the inclusion criteria, and 2 opted not to participate. A total of 30 remaining participants were randomly distributed across two equal GPs, with 15 participants in each GP. All participants ended follow-up and were incorporated into the final statistical analysis Figure 2.

There was no statistically notable variation between the two GPs in terms of demographic characteristics and medical history. Regarding fracture patterns, the simpler fracture type (AO/OTA 31-A2) was the most prevalent in both GPs, with an incidence of 53.3% in GP A and 80% in GP B Table 1.

**Table 1:** Comparison between two GPs regarding demographic data, medical history, and fracture assessment.

		GP A (LCMN) (*n* = 15)	GP B (SCMN) (*n* = 15)	Test	*P*
Age (years)		72.33 ± 10.11	71.07 ± 8.59	*t* = 0.37	0.71
Sex	Male	3 (20.0%)	5 (33.3%)	FE = 0.68	0.68
	Female	12 (80.0%)	10 (66.7%)		
Occupation	Working	5 (33.3%)	2 (13.3%)	FE = 1.68	0.39
**Medical history**					
Level of activity pre-injury	Unassisted	10 (66.7%)	11 (73.3%)	FE = 0.16	1.00
	Assisted	5 (33.3%)	4 (26.7%)		
Mechanism of injury	Fall	12 (80.0%)	12 (80.0%)	FE = 0.00	1.00
	RTA	3 (20.0%)	3 (20.0%)		
Smoking		1 (6.7%)	2 (13.3%)	FE = 0.37	1.00
HTN		9 (60.0%)	8 (53.3%)	*X*^2^ = 0.14	0.71
DM		6 (40.0%)	6 (40.0%)	*X*^2^ = 0.00	1.00
Cardiac		6 (40.0%)	2 (13.3%)	FE = 2.73	0.22
Others		1 (6.7%)	2 (13.3%)	FE = 0.37	1.00
Comorbidities		11 (73.3%)	13 (86.7%)	FE = 0.83	0.65
**Fracture assessment**					
NSA (degrees)		114.13 ± 8.43	108.53 ± 7.28	*t* = 1.95	0.06
Lateral wall thickness (mm)		17.61 ± 8.06	20.37 ± 7.72	*t* = 0.94	0.36
Canal diameter at the isthmus (mm)		8.07 ± 0.70	8.40 ± 0.51	*t* = 1.49	0.15
Fragment size distal to base of LT (mm)		11.00 ± 4.31	7.30 ± 3.43	*t* = 2.03	0.06
HHS pre-injury		88.80 ± 8.71	87.53 ± 14.14	*t* = 0.30	0.77
BI pre-injury		91.33 ± 10.93	96.67 ± 6.17	*t* = 1.65	0.11
Injury side	Right	8 (53.3%)	5 (33.3%)	*X*^2^ = 1.22	0.27
	Left	7 (46.7%)	10 (66.7%)		
AO classification	31-A2.2	6 (40.0%)	7 (46.7%)	FE = 3.84	0.50
	31-A2.3	2 (13.3%)	5 (33.3%)		
	31-A3.1	2 (13.3%)	0 (0.0%)		
	31-A3.2	1 (6.7%)	1 (6.7%)		
	31-A3.3	4 (26.7%)	2 (13.3%)		
AO classification	31-A2	8 (53.3%)	12 (80.0%)	*X*^2^ = 2.40	0.12
	31-A3	7 (46.7%)	3 (20.0%)		
Evans classification	Type 1 GP 3	5 (33.3%)	9 (60.0%)	FE = 6.10	0.05
	Type 1 GP 4	5 (33.3%)	6 (40.0%)		
	Type 2	5 (33.3%)	0 (0.0%)		
Evans classification	Type 1	10 (66.7%)	15 (100.0%)	FE = 6.00	0.04*
	Type 2	5 (33.3%)	0 (0.0%)		
MHHS pre	Excellent	8 (53.3%)	9 (60.0%)	FE = 3.04	0.39
	Good	5 (33.3%)	2 (13.3%)		
	Fair	2 (13.3%)	2 (13.3%)		
	Poor	0 (0.0%)	2 (13.3%)		

Data is shown as mean ± SD or frequency (%). *t*: Student *t* test, FE: Fisher Exact, *X*^2^: Chi square test. RTA: Road traffic accident, HTN: hypertension, DM: diabetes mellitus, NSA: neck shaft angle, LT: lesser trochanter, HHS: Harris hip score, MHHS: modified HHS, BI: Barthel index. Bold values represent *p* value less than 0.05.

The majority of the patients in the long group reported a fair HHS score at 3 months compared to 5 patients in the short group. Regarding the BI score, an average of at least 15 more points was recorded more than the 3 weeks observed values. Eight patients (57.1%) in group A and seven patients (50%) in group B had good to excellent HHS scores at the time of the last follow-up, with an average of 76.86 ± 10.77 and 80.08 ± 11.21, respectively. Both groups had an average BI of at least 85 points Table 2.

**Table 2:** Comparison between two GPs regarding operative data and at 3 weeks, 3, and 6 months follow-up.

		GP A (*n* = 15)	GP B (*n* = 15)	Test	*P*
Duration from admission to procedure (days)		4.60 ± 2.95	4.33 ± 2.47	*t* = 0.27	0.79
Total duration of hospital stays (days)		7.64 ± 2.50	7.00 ± 2.96	*t* = 0.62	0.54
Nail length (mm)		369.33 ± 14.86	194.67 ± 14.07	*t* = 33.05	<0.001*
Nail width (mm)		11.60 ± 0.51	11.87 ± 0.35	*t* = 1.67	0.11
TAD (mm)		23.27 ± 7.00	19.43 ± 5.59	*t* = 1.66	0.11
Operative time (minutes)		93.00 ± 17.09	85.67 ± 25.35	*t* = 0.93	0.36
Estimated blood loss (ml)		236.67 ± 229.49	140.00 ± 92.97	*t* = 1.51	0.14
Anti-rotation screw vs endcap	Antirotation screw	12 (80.0%)	10 (66.7%)	FE = 0.68	0.68
	Endcap	3 (20.0%)	5 (33.3%)		
Anesthesia	Spinal	14 (93.3%)	15 (100.0%)	FE = 1.03	1.00
	General	1 (6.7%)	0 (0.0%)		
Reduction	Closed	11 (73.3%)	14 (93.3%)	FE = 2.16	0.33
	Open	4 (26.7%)	1 (6.7%)		
Blood transfusion	No	8 (53.3%)	13 (86.7%)	FE = 3.97	0.11
	Yes	7 (46.7%)	2 (13.3%)		
**At 3 weeks of follow-up**					
HHS		47.00 ± 15.13	42.79 ± 13.30	*t* = 0.78	0.44
BI		57.86 ± 17.84	53.00 ± 15.08	*t* = 0.78	0.44
NSA		130.36 ± 5.00	128.50 ± 3.80	*t* = 1.05	0.30
Time to weight bear (partial)	Within 2 weeks	7 (46.7%)	5 (33.3%)	FE = 1.69	0.77
	2–4 weeks	3 (20.0%)	6 (40.0%)		
	>4 weeks	3 (20.0%)	2 (13.3%)		
	Never	2 (13.3%)	2 (13.3%)		
Union	No	14 (100.0%)	14 (92.9%)	–	–
	Yes	0 (0.0%)	0 (7.1%)		
HHS	Fair	3 (20.0%)	1 (6.7%)	FE = 1.15	0.60
	Poor	12 (80.0%)	14 (93.3%)		
**At 3 months follow-up**					
HHS		67.36 ± 9.72	66.15 ± 11.44	*t* = 0.30	0.77
BI		75.57 ± 13.98	76.54 ± 11.44	*t* = 0.20	0.85
NSA		130.14 ± 5.56	126.86 ± 6.07	*t* = 1.49	0.15
Union	No	3 (21.4%)	1 (0.0%)	FE = 1.167	0.60
	Yes	11 (78.6%	13 (92.86%)		
HHS	Excellent	0 (0.0%)	0 (0.0%)	FE = 1.43	0.56
	Good	1 (7.1%)	2 (14.3%)		
	Fair	8 (57.1%)	5 (35.7%)		
	Poor	5 (35.7%)	7 (50.0%)		
**At 6 months follow-up**					
HHS		76.86 ± 10.77	80.08 ± 11.21	*t* = 0.76	0.45
BI		85.00 ± 14.68	90.77 ± 10.38	*t* = 1.17	0.25
NSA		130.14 ± 5.56	128.00 ± 3.98	*t* = 1.14	0.26
Union	No	0 (0.0%)	1 (7.69%)	FE = 1.04	1.00
	Yes	14 (100.0%)	13 (92.86%)		
HHS	Excellent	1 (7.1%)	2 (14.3%)	FE = 1.02	0.90
	Good	7 (50.0%)	5 (35.7%)		
	Fair	4 (28.6%)	5 (35.7%)		
	Poor	2 (14.3%)	2 (14.3%)		

Data is presented as mean ± SD or frequency (%). *Significant *P* value (in bold) < 0.05. *t*: Student’s *t*-test, FE: Fisher’s Exact. TAD: tip apex distance, HHS: Harris hip score, NSA: neck shaft angle, BI: Barthel index.

### Complications

**Figure 3:**
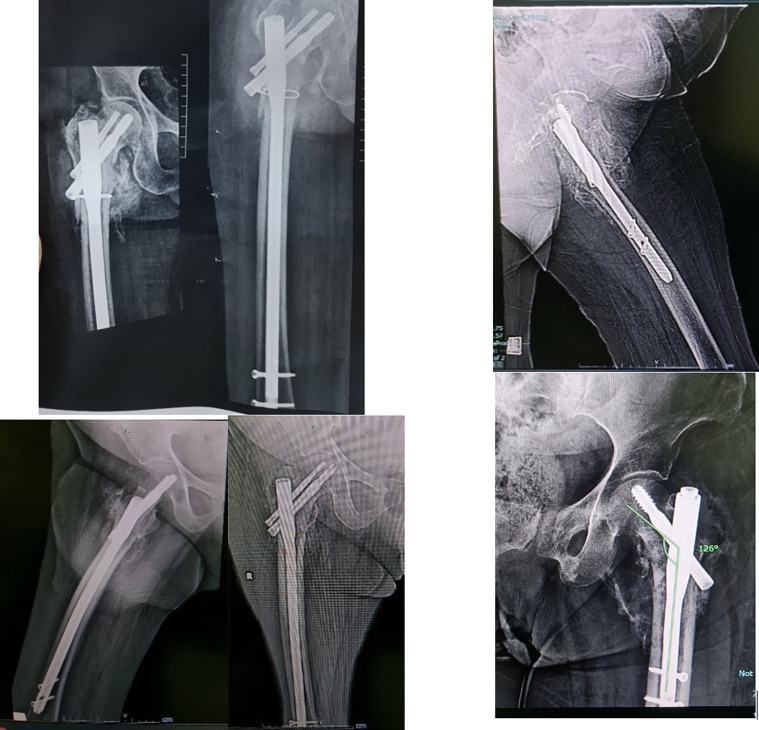
Heterotrophic ossification.

During the 6-month follow-up period, 1 case died in each group within the first 35 days during their hospital stay due to poor general condition. From the LCMN, 1 patient was diagnosed with FRI within the first 3 weeks, which was managed by DIAR (debridement, irrigation, and implant retention) and later by antibiotic suppression till signs of union were observed at 3 months. In Group A, 2 patients complained of hip pain and had radiological signs of HO compared to 1 patient in the short group. They were prescribed (NSAIDS) indomethacin for 1 month and reported pain improvement Figure 3.

**Figure 4:**
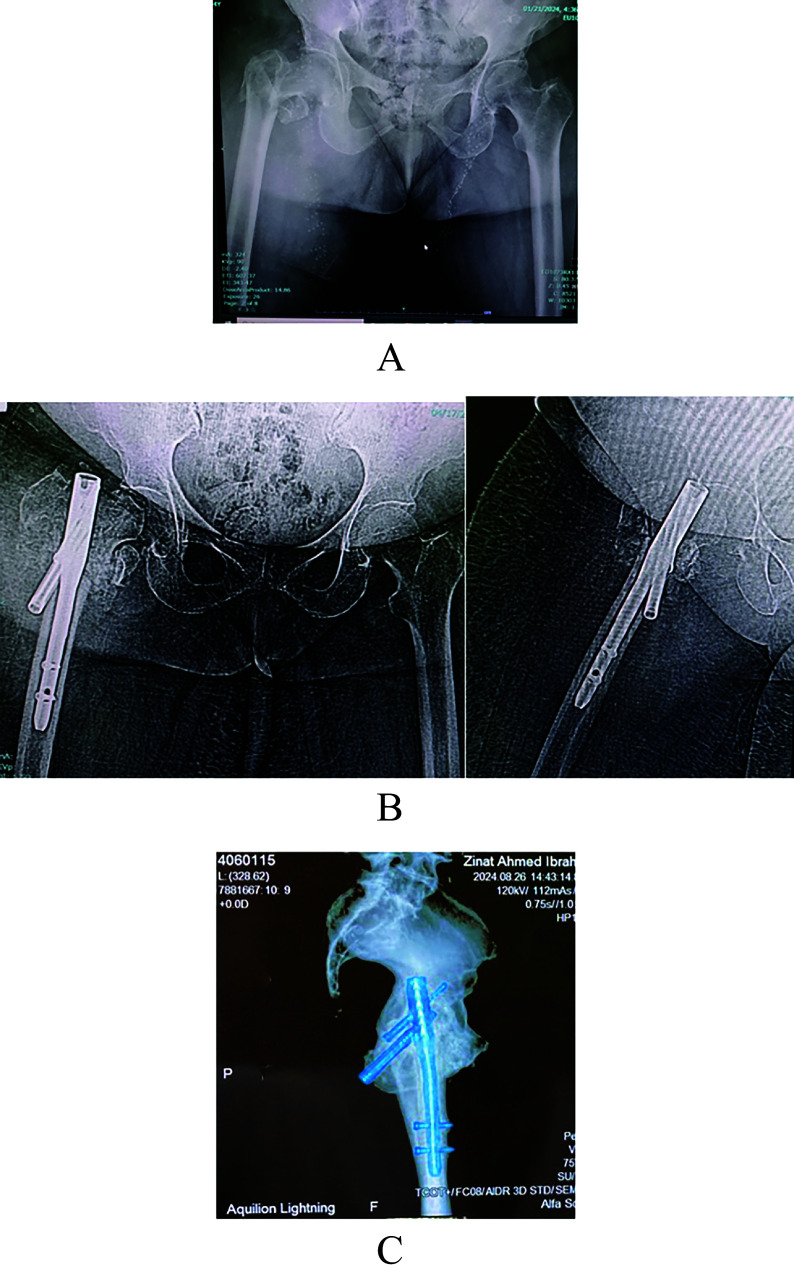
(A) (AO 31–A2.3) fracture pattern, (B) AP and lateral views 2 m later post-surgery showing AVN and varus collapse, and (C) CT 3D showing z effect.

Among the short GP, a patient presented with implant failure at a 2-month interval with avascular necrosis of the femoral head. There was significant varus collapse (>5°) with a z effect. Patient underwent implant removal and arthroplasty Figure 4.

**Figure 5:**
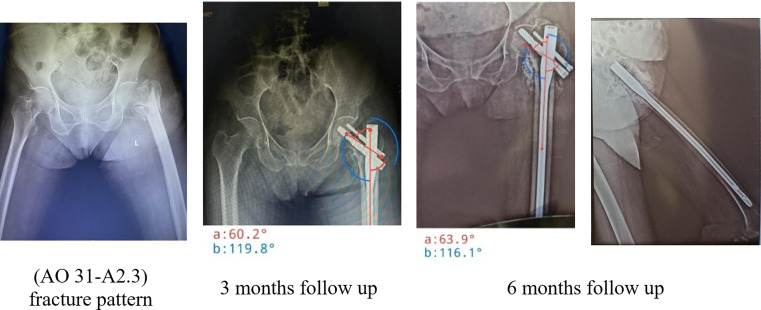
Long CMN varus collapse and screw back out.

Two more patients in the short GP had varus collapse with the screw back out. One of which had additional screw breakage; both underwent implant removal.Only one patient in the long GP presented with varus collapse and screw cut-out Figure 5.

Despite a higher complication rate with short CMN, no significant difference was found between groups Table 3.

**Table 3:** Comparison between two GPs regarding the rate of complications.

	GP A (*n* = 15)	GP B (*n* = 15)	Test	*P*
Mortality	1 (6.6%)	1 (6.6%)	*X*^2^ = 3.624	0.057
Varus collapse (>5°)	2 (13.3%)	3 (20.0%)		
Screw back out	2 (13.3%)	3 (20.0%)		
Screw cut out	1 (6.6%)	1 (6.6%)		
Implant failure and AVN	0 (0.0%)	1 (6.6%)		
HO	2 (13.3%)	1 (6.6%)		
FRI	1 (6.6%)	0 (0.0%)		
Radiographic nonunion	0 (0.0%)	1 (6.6%)		
Chronic anterior thigh pain	2 (13.3%)	3 (20.0%)		

Data is shown as frequency (%). *X*^2^: Chi-square test. AVN: avascular necrosis, HO: heterotopic ossification, FRI: fracture-related infection.

## Discussion

ITF are extracapsular fractures located in the proximal femur extending between the GT and LT [1].The most frequent mechanism of trauma was a trivial fall (80%), coinciding with the results produced by a similar RCT by Rahman et al. [12]. This was pointed out by the fact that 24 patients (80%) in this study had at least one co-morbidity, with 10 patients from both GPs being polymorbid, contributing to the fall and subsequent fracture.

As the most recent 2018 revision (AO/OTA) fracture and dislocation classification compendium reviewed, unstable ITF includes 31-A2.2, 31-A2.3, 31-A3.1, 31-A3.2, and 31-A3.3. The previous AO 31-A2.1 classification is left blank in the current classification and is no longer considered a valid secondary classification criterion due to the questionable contribution of LT to the stability [13]. That being said, Gotfried et al. [14] and Pradeep et al. [15] highlighted the relevance of the lateral wall integrity regarding the implant choice and therapeutic outcome. After traction x-rays, the majority of patients were presented with the simpler pattern AO 31-A2, and classified by Evans into type 1 fractures (with subtypes GP 3 and 4).

The total duration of hospital stay was comparable in both GPs. Nevertheless, the mean average time of operation was higher in the long CMN GP, possibly due to the nature of the procedure, where serial whole-length medullary reaming is required and the trials needed to place the distal screws ideally into their nail holes using the perfect circle technique or aiming jig with image fluoroscopy assistance. On the contrary, SCMN had a lower mean operative time as only partial medullary reaming is needed, and an aiming device is available for distal locking. Despite those mean differences, no significant differences were found between the two GPs compared to Boone et al. [16] where their results established a significant difference between the two GPs.

The mean estimated blood loss (236.67 mL) and transfusion necessity were higher in the long CMN GP, possibly due to the higher mean operative surgical time and the need for open reduction in those patients, as nearly half of the patients (7) had the more complex AO-31-A3 fracture subtype.

About two-thirds of the patients in both GPs were able to bear partial weight within 1 month, with an earlier proportion of mobile patients in the long GP with a ratio (1.4:1) within 2 weeks. At the final follow-up period, all patients achieved union apart from one patient in the short GP, who presented with implant failure, AVN and need for revision surgery. More than or equal to 50% of GPs achieved a good to excellent HHS score and above 80 Barthel scores. No marked difference was found between both GPs in terms of the functional outcome, concluding that both implants are valid options for unstable intertrochanteric fractures. Similar conclusions were reached by Ocku et al. [17].

No periprosthetic fractures were documented in our research, probably due to the time-dependent nature of this complication since the index procedure and all nails were locked distally, which may offer a protective advantage against refractures as portrayed by the retrospective study of Lindvall et al. [18] study.

The overall complication rate between both GPs was insignificant.

The limitations of the research include a relatively restricted sample population with a limited follow-up period, which may affect the validity and longitudinal relevance of the findings. Since it’s a surgical procedure, double blinding was not possible as the surgeon was exposed to the nail dimensions. The study was retrospectively registered in PACTR.

Irrespective of the length, CMN are suitable tools for the treatment of unstable ITF to achieve early pain-free mobility and functional outcome analogous to that of the pre-injury level. Despite higher mean values of total hospital stay, period of surgery, intraoperative blood loss, and blood transfusion in the LCMN, results were comparable regarding union, complication rates, and functional outcomes with no significant differences between the two groups. It’s advised to treat ITF on a case-by-case basis due to the heterogeneity of fracture patterns. It’s crucial to weigh the choice of implant; with a tendency to use SCMN in the elderly with a feeble nature to cut off operative time and blood loss relative to LCMN, which spans the femur, adding a biomechanical advantage in osteoporotic bones. We recommend larger randomized trials comparing short and long CMN, using modern fracture classifications (Kalia and Singh CT) and incorporating the Singh index to guide nail selection [19].

## Data Availability

Data are available from the corresponding author upon request.
